# Supplementary material for: Patterns of Intron Gain and Loss in Fungi
Source: PLoS Biol. 2004 Nov 30;2(12):e422. doi: 10.1371/journal.pbio.0020422 (PMC532390; doi:10.1371/journal.pbio.0020422)
Supplement: Table S1 — Also available at http://genes.mit.edu/NielsenEtAl/. (4.3 MB ZIP). [file pbio.0020422.st001.zip › NielsenEtAl/html/8.html]

AN7305.1.NCU08668.1.MG05815.1.FG09761.1


```
 CLUSTAL W (1.82) Multiple Sequence Alignments - Introns Inserted


Sequence 1: NCU08668.1	340 aa
Sequence 2: FG09761.1	321 aa
Sequence 3: MG05815.1	330 aa
Sequence 4: AN7305.1	346 aa
Alignment Length: 360 aa
Number Identitical Residues: 83 aa
Alignment Score (without introns) 4817


MG05815.1 	MAP-EKRNHFLDADESDD-DQSHGYNSEAEELTKGGRSAKRRKIGS-----DSDVDDDDD
NCU08668.1	MPEDDVRNKFLDAGDSDD-DAGHGSDSE-DDFQKGGRNAKRRRVSD-----DEDSEADDF
FG09761.1 	MAP-ETKNDFLEIADSDA-DSDIGYNSEEDDMQKGGRGAKRRKVES-----DDE---DDP
AN7305.1  	-MTTRKHNEFLDIGTSDDEDNDAGYDSEAAEESKG-RTVKRRKTQSREDGIESASEDESS
          	      :*.**:   ** .* . * :**  :  ** * .***:  . ... :.  . :. 

MG05815.1 	FSDQEDDNSALSPTQQLQDEDGASARDGAKGGDEGDGATSRPKKSKDAGPKLRTDAANPL
NCU08668.1	TDAEEEHDQDDAESKDAPAKDGQETTDGKEKKDG-----KKEKEKKSVLASDLPGMTKPL
FG09761.1 	GSDIGSDDEDEATEKDS--KKTQEE-DKKEDQPE-----KPKRKSKDTTSTELPDVTRTL
AN7305.1  	GGSEGEESDTEAGGVRLREKKRGQRDEYENDSDDDGNYMNQESQYLDVTAEVEKAKAKKN
          	 .   ....  :       :.  .  :  :    ...  .   :  .. .      :.  

MG05815.1 	-KKNLVVTDKQIKK--SGVVYLSRIPPFMKPSKLRSLLVP--YGKINRIFLSPEDPEARK
NCU08668.1	TKKNLIVTEEAIKK--SGVVYISRVPPFMTPAKLRSLLEP--YGKLNRIFLAPEDPVARR
FG09761.1 	TKKNLVASEAAIKK--SGVVYLSRIPPFMKPAKLRSLLEP--YGTINRIFLAPEDPASHA
AN7305.1  	QKPLDKLKNKPPKKNKTGVVYFSSLPPYLKPFALKSLLETRGFKPITKVFLTPAVRPPSA
          	 *     .:   **..:****:* :**::.*  *:*** . .:  :.::**:*    .  

MG05815.1 	RRLKAGGNKKRCFVDGWVEFMDKKD~AKAVAEVLNGQTIGGKKGSYYRDD~IWTLLYLKG
NCU08668.1	KRIRSGGNKKKMFTEGWIEFVKKKD1AKKACELLNARPIGGKKGSYYRDD~IWNLLYLKG
FG09761.1 	RRVRAGGNKKRSYTEGWVEFTKKKD~AKAVCDLLNARTIGGKKGSYYHDD~LWNLLYLKG
AN7305.1  	PRRRS--NKRKTYTDGWVEFASKKT~AKICAETLNATIVGGKKGGWYHDD1VWNMKYLKG
          	 * ::  **:: :.:**:** .**  **  .: **.  :*****.:*:** :*.: ****

MG05815.1 	FKWHNLMEQIAAENLERANRMRAEIARSTKENKEFVRQAERAKVLEGIQSKKKAKGQGEE
NCU08668.1	FKWHNLTEQIAAENAERSSRMRAEISKTTKENKEFVRNVERAKVLQGIQAKKASKGSKAG
FG09761.1 	FKWHNLTEQIAAENAERSSRMRAEISKSTKENKEFVRNVEKAKMLDGMAVKAKAKKRKAE
AN7305.1  	FKWADLMETQERERSEREAKRRIEDARARKEEKVFLQGYEKGKMLEGIQKKNEEKRRKKM
          	*** :* *    *. **  : * * ::: **:* *::  *:.*:*:*:  *   *     

MG05815.1 	VENAQNDGGAVK-------------SRSMKFEQVHVASKKE----TTEP-PAQAQKTLRT
NCU08668.1	GEGAAQVTESTIPSAAATTTTTTNDDKRRTFKQIPLAKKRK----LDETQPEQVQRVLSK
FG09761.1 	THTEGQEDESVR-------------QVKRSFKQVPLAKKKM----DGEDQPAEVTRVLSK
AN7305.1  	ADKDGHGDQGKE----------QAINARRLFKQNEVKLGRDKTSADQTTVEEDTRRVLGK
          	 .   :   .               .    *:*  :   : .:::       :. :.* .

MG05815.1 	LF
NCU08668.1	IF
FG09761.1 	IF
AN7305.1  	IF
          	:*
```
